# Protective effect of resin adsorption on septic plasma-induced tubular injury

**DOI:** 10.1186/cc8835

**Published:** 2010-01-11

**Authors:** Vincenzo Cantaluppi, Viktoria Weber, Carola Lauritano, Federico Figliolini, Silvia Beltramo, Luigi Biancone, Massimo De Cal, Dinna Cruz, Claudio Ronco, Giuseppe Paolo Segoloni, Ciro Tetta, Giovanni Camussi

**Affiliations:** 1 Center for Experimental Medical Research (CeRMS), University of Torino, Via Santena 5, Torino 10126, Italy; 2 Nephrology, Dialysis and Renal Transplantation Unit, Department of Internal Medicine, University of Torino, Corso Dogliotti 14, 10126, Italy; 3 Department for Clinical Medicine and Biotechnology, Center for Biomedical Technology, Danube University Krems, Dr. Karl Dorrek Street 30, Krems, A-3500, Austria; 4 Department of Nephrology, Dialysis and Transplantation, San Bortolo Hospital, Viale Rodolfi 37, Vicenza, 36100, Italy; 5 Fresenius Medical Care, Daimlerstrasse 15, Bad Homburg, D-61352, Germany; 6 Cattedra di Nefrologia, Dipartimento di Medicina Interna, Ospedale Maggiore S. Giovanni Battista, Corso Dogliotti 14, 10126, Torino, Italy

## Abstract

**Introduction:**

A pro-apoptotic effect of circulating mediators on renal tubular epithelial cells has been involved in the pathogenesis of sepsis-associated acute kidney injury (AKI). Adsorption techniques have been showed to efficiently remove inflammatory cytokines from plasma. The aim of this study was to evaluate the efficiency of the hydrophobic resin Amberchrom CG161 M to adsorb from septic plasma soluble mediators involved in tubular injury.

**Methods:**

We enrolled in the study 10 critically ill patients with sepsis-associated AKI and we evaluated the effects of their plasma on granulocyte adhesion, apoptosis and functional alterations of cultured human kidney tubular epithelial cells. We established an *in vitro *model of plasma adsorption and we studied the protective effect of unselective removal of soluble mediators by the Amberchrom CG161 M resin on septic plasma-induced tubular cell injury.

**Results:**

Plasma from septic patients induced granulocyte adhesion, apoptosis and altered polarity in tubular cells. Plasma adsorption significantly decreased these effects and abated the concentrations of several soluble mediators. The inhibition of granulocyte adhesion to tubular cells was associated with the down-regulation of ICAM-1 and CD40. Resin adsorption inhibited tubular cell apoptosis induced by septic plasma by down-regulating the activation of caspase-3, 8, 9 and of Fas/death receptor-mediated signalling pathways. The alteration of cell polarity, morphogenesis, protein reabsorption and the down-regulation of the tight junction molecule ZO-1, of the sodium transporter NHE3, of the glucose transporter GLUT-2 and of the endocytic receptor megalin all induced by septic plasma were significantly reduced by resin adsorption.

**Conclusions:**

Septic plasma induced a direct injury of tubular cells by favouring granulocyte adhesion, by inducing cell apoptosis and by altering cell polarity and function. All these biological effects are related to the presence of circulating inflammatory mediators that can be efficiently removed by resin adsorption with a consequent limitation of tubular cell injury.

## Introduction

The incidence of acute kidney injury (AKI) has considerably increased during the past few years [1,2]. AKI is a frequent complication occurring in critically ill patients with sepsis or septic shock [3-5]. The mechanisms of sepsis-induced tissue injury are complex and seem to be related not only to the ischemic response to hypoperfusion, but also to a direct detrimental activity induced by circulating mediators with both pro- and anti-inflammatory properties able to interact in a dynamic manner and to induce multiple organ failure [5,6].

We recently showed that plasma derived from septic patients with severe burns induced apoptosis and functional alterations of glomerular podocytes and tubular epithelial cells (TEC) [7]. These data confirmed the observations coming from different studies showing that inflammatory cytokines and lipopolysaccharides (LPS) activated the apoptotic pathways in tubular cells via caspase activation and Fas up-regulation [8-10]. In addition, in experimental animal models of sepsis, a broad range of functional alterations of tubular re-absorption such as sodium, urea and glucose renal transporter dysfunction has been reported in the presence of an inflammatory microenvironment [11-13]. Taken together, these data support the hypothesis of a prominent role of circulating mediators in the pathogenesis of sepsis-related AKI.

Renal replacement therapy (RRT) is an important therapeutic strategy in patients with AKI. Several studies suggested that RRT is able to maintain adequate fluid, electrolyte and acid-base balance but can also favorably influence the outcome of AKI patients by removing a broad range of inflammatory substances [14-16]. Various mechanisms have been proposed for such removal: diffusion, convection and adsorption [17]. Indeed, the adsorption matrixes may be useful tools to remove different inflammatory mediators by non-selective simultaneous adsorption [18,19]. Based on previous studies, Amberchrom CG161 M, a rigid, highly cross-linked microreticular hydrophobic adsorbent polymer was chosen as having the most convenient particle and pore size [20].

The aim of this study was to establish an *in vitro *model of tubular injury based on the effects of septic plasma and to evaluate whether the unselective removal of circulating plasma factors by the Amberchrom resin could be protective on septic plasma-induced tubular cell injury.

## Materials and methods

### Patients

From June to December 2008, 10 critically ill patients (mean age: 63.9 ± 11.2 years; gender: seven males, three females) admitted to the intensive care unit (ICU) of the San Bortolo Hospital in Vicenza, Italy, were enrolled in the study. Inclusion criteria were: the presence of septic shock in accordance to the criteria defined by the American College of Chest Physicians and by the Society of Critical Care Medicine [21]; and the presence of AKI determined by the evaluation of serum creatinine or urinary output (inclusion in the failure group of RIFLE criteria) [22,23]. Exclusion criteria were: age younger than 18 years, solid organ or bone marrow transplantation, hemorrhagic dysfunction, thrombophilia, chronic renal failure, glomerulonephritis or collagenopathies. The severity of illness was assessed by Sequential Organ Failure Assessment (SOFA) score at the moment of ICU admission and at the start of the dialytic treatment. As control, plasma was obtained from five healthy volunteers. Informed consent was obtained according to the Declaration of Helsinki and the study was authorized by the Internal Review Board of the San Bortolo Hospital.

### *In vitro *plasma adsorption: experimental design

The Amberchrom CG161 M resin (Rohm and Haas Company, Philadelphia, PA, USA). was activated in 50% methanol and extensively washed in isotonic saline. Two ml of the resin were packed into chromatography columns with an inner diameter of 1 cm (Biorad, Hercules, CA, USA). Prior to filling with the resin, columns were treated with silane (Sigma, St. Louis, MO, USA). The resin beds were perfused with a solution of 4% human serum albumin in PBS containing a cocktail of recombinant cytokines at the following concentrations (pg/ml): TNF-α (600), IL-1β (200), IL-10 (350), IL-8 (400), and IL-6 (300). For IL-1β, an additional series of experiments was carried out using 1 ml of adsorbent and an IL-1β spike concentration of 300 pg/ml. The flow rate was set to 0.3 ml/min corresponding to a linear velocity of 22 cm/h. Fractions of 2 ml were collected and stored at -80°C until assayed (see below). Before *in vitro *tests on tubular cells, the Amberchrom CG161 M resin was extensively washed by isotonic saline and then mixed with plasma collected from patients with sepsis-related AKI (90% volume plasma + 10% volume Amberchrom CG161 M resin). Plasma/resin mixture was kept in a condition of slight agitation at 37°C for 120 minutes. Samples were taken in sterile conditions after 15, 30, 60 and 120 minutes of agitation. At the start and at the end of adsorption, plasmatic levels of TNF-α, Fas-Ligand (Fas-L) and CD40-Ligand (CD40-L or CD154) were determined by ELISA (R&D Systems, Minneapolis, MN, USA). Results were calculated after generation of a standard curve with appropriate controls and given as averages ± standard deviation (SD).

### Isolation and characterization of human proximal tubular epithelial cells and umbilical vein endothelial cells

Primary cultures of human proximal TEC were obtained from kidneys removed by surgical procedures from patients affected by renal carcinomas as previously described [24]. Primary TEC were immortalized by infection with a hybrid Adeno5/SV40 virus [25] and cultured with RPMI 1640 (GIBCO, Grand Island, NY, USA) containing 10% FCS (Hyclone, Logan, UT, USA) and 2 mM glutamine (GIBCO, Grand Island, NY, USA). The purity of TEC cultures was assessed on the basis of cell characterization, according to published criteria [24,25]. Human umbilical vein endothelial cells (HUVEC) were isolated and characterized as previously described [26].

### Adhesion of polymorphonuclear neutrophils to TEC or HUVEC monolayers

Polymorphonuclear neutrophils (PMN) were isolated from blood of healthy volunteers by density centrifugation as previously described [27] and labeled overnight with 10 μM Vybrant Cell Tracer kit (Invitrogen, San Diego, CA, USA) according to the manufacturer's instructions in RPMI and 10% FBS. Labeled cells were counted, resuspended to 50 × 10^6^/ml RPMI and added to a confluent monolayer of TEC or HUVEC cultured on six-well plates and previously incubated with different plasma samples. Experiments were carried out in triplicate for one hour at 37°C in conditions of slight agitation. At the end of incubation, plates were filled with medium and aspirated three times to remove unbound cells. All samples were fixed with 1% paraformaldehyde and observed under a UV light microscope. Green fluorescent cells were counted on 10 different fields at ×100 magnification.

### Cytotoxicity assay

TEC were cultured on 24-well plates (Falcon Labware, Oxnard, CA, USA) at a concentration of 5 × 10^4 ^cells/well and incubated with different plasma concentrations and 250 μg/ml XTT (Sigma, St. Louis, MO, USA) in a medium lacking phenol red. The absorption values at 450 nm were measured in an automated spectrophotometer at different time points. All experiments were performed in triplicate.

### Detection of apoptosis

#### TUNEL assay

TEC were incubated with different plasma and then subjected to terminal deoxynucleotidyltransferase-mediated dUTP nick end labelling (TUNEL) assay (ApopTag, Oncor, Gaithersburg, MD, USA) that identifies DNA fragmentation, a typical feature of apoptotic cells. Green-stained apoptotic cells were counted in different microscopic fields at ×100 magnification. In selected experiments, LPS (30 ng/ml) (Sigma, St. Louis, MO, USA), polymyxin B (5 μg/ml) (Sigma, St. Louis, MO, USA), TNF-α (20 ng/ml) (Sigma, St. Louis, MO, USA) and interferon (IFN)-γ (20 ng/ml) (Sigma, St. Louis, MO, USA) were used.

#### Propidium iodide nuclear staining

Propidium iodide nuclear staining was used to identify DNA fragmentation, a typical feature of apoptotic cells. TEC were cytospinned, fixed with 1% paraformaldehyde and stained by a solution containing 50 μg/ml propidum iodide (Sigma, St. Louis, MO, USA), 0.1% sodium citrate (Sigma, St. Louis, MO, USA), 0.1% Triton-X-100 (Sigma, St. Louis, MO, USA) and 20 μg/ml DNase-free RNase (Sigma, St. Louis, MO, USA) diluted in sterile water. All samples were examined by UV light microscopy.

### Generation of transfectants and RNA interference

Chinese hamster ovary (CHO) transfectants were generated by electroporation with plasmid vectors containing cDNA coding for a soluble form of Fas-L, CD40-L (gp39-CD8) or with control empty vectors (mock) at 250 V and 960 μF in 4 mm electroporation cuvettes in an electroporator II (Invitrogen, San Diego, CA, USA). Clones were selected for 1 mg/ml G418 resistance in RPMI 1640 plus 10% FCS. After selection, supernatants were collected and used for *in vitro *tests on TEC.

In selected experiments TEC were seeded on six-well plates and TNF-receptor (R) 1, Fas, CD40 siRNA or relative control siRNA (80 pM) was introduced according to manufacturer's instructions (Santa Cruz Biotech, Santa Cruz, CA, USA). After 48 hours, the effective suppression of specific mRNAs and proteins was verified by RT-PCR and by immunofluorescence or western blot analysis. Subsequently, engineered cells were used to evaluate plasma-induced apoptosis and PMN adhesion.

### Caspases-3, -8 and -9 activity

The activity of caspase-3, -8 and -9 was assessed by a colorimetric assay (Chemicon Int., Temecula, CA, USA) based on the spectrophotometric detection of the cromophore p-nitroanilide (pNA) after cleavage from the labelled substrate DEVD-pNA by caspases [24]. Each experiment was performed in triplicate. Results are given as average of percentage increase of caspase activity in respect to incubation with control healthy plasma ± SD.

### Analysis of transepithelial electrical resistance

Transepithelial electrical resistance (TER) was used as an indicator of TEC polarity. Cells were plated in transwells on collagen-coated polycarbonate membranes (Corning Costar Corp., Cambridge, MA, USA) and allowed to reach confluence before the addition of different plasma samples. An epithelial volt-ohm meter (EVOM; World Precision Instruments, Inc., Sarasota, FL, USA) was used to determine TER values as previously described [24]. All measures were performed in triplicate and normalized for the area of the membrane.

### Tubular adhesion to extracellular matrixes and morphogenesis assay

Adhesion of TEC to extracellular matrixes was evaluated on 24-well culture plates previously coated for six hours with 20 μg/ml of human fibronectin/type IV collagen or Matrigel (Becton Dickinson, Franklin Lakes, NJ, USA). Non-specific adhesion was blocked by incubation for two hours with 2% BSA diluted in one times PBS. TEC were exposed to different plasma for six hours at 37°C in conditions of slight agitation. Thereafter, aliquots of stimulated cells were added to the wells and allowed to adhere for two hours at 37°C. Supernatants were then removed and attached cells were subjected to the XTT-based assay as reported above. For morphogenesis studies, TEC were cultured on Matrigel-coated plates for 72 hours and in the presence of control healthy or septic plasmas.

### Detection of FITC-conjugated albumin uptake

Albumin uptake was studied after incubation of TEC previously exposed to different plasma with 50 mg/ml of FITC-conjugated human albumin (Sigma, St. Louis, MO, USA) at 37°C for two hours. After FITC-albumin challenge, TEC were extensively washed twice with ice-cold one times PBS and analysed by FACS and confocal microscopy after co-staining with an antibody directed to megalin (see below).

### Immunofluorescence studies

After appropriate stimuli, cultured TEC were fixed in ethanol/acetic acid 2:1 and stained with antibodies directed to human Fas (Upstate Biotechnology, Lake Placid, NY, USA), zonula occludens-1 (ZO-1), megalin, proximal tubular sodium transporter sodium-hydrogen exchanger-3 (NHE3) and glucose transporter 2 (GLUT-2; Santa Cruz Biotech, Santa Cruz, CA, USA). After incubation with primary antibodies, samples were washed with one times PBS and incubated with appropriated Alexa Fluor-conjugated secondary antibodies (Molecular Probes, Carlsbad, CA, USA) for 30 minutes, room temperature when needed. All samples were counterstained by 1 μg/ml propidium iodide or 0.5 μg/ml Hoechst for 30 seconds, mounted with anti-fade mounting medium (Vector Laboratories, Burlingame, CA, USA) and examined by confocal microscopy.

### FACS analysis

For FACS analysis, after exposure to different plasmas, TEC were detached from tissue culture plates with EDTA, washed twice with one times PBS and stained for one hour at 4°C with FITC-conjugated antibodies directed to human Fas, CD40, inter-cellular adhesion molecule-1 (ICAM-1) (Becton Dickinson, Franklin Lakes, NJ, USA) or with an irrelevant control antibody. All incubation periods were performed using a medium containing 0.25% BSA and 0.0016% sodium azide. At the end of staining, cells were newly washed, fixed in 1% paraformaldehyde and subjected to FACS analysis (Becton Dickinson, Franklin Lakes, NJ, USA).

### Statistical analysis

All data of different experimental procedures are expressed as average ± SD. Statistical analysis was performed by analysis of variance with Newmann-Keuls multicomparison test or Student's *t-*test where appropriated. The *P *values less than 0.05 were considered as the threshold for significance.

## Results

### Patients

Selected patients with sepsis-associated AKI showed an average SOFA score of 13.4 ± 7.1 at the start of the dialytic treatment. AKI was detected by the rise of serum creatinine (3.3 ± 1.6 mg/dl) and urea (146 ± 84.7 mg/dl). All patients were included in the failure group of RIFLE criteria [23].

### Effect of plasma adsorption on PMN-TEC and PMN-HUVEC interaction

**Figure 1:**
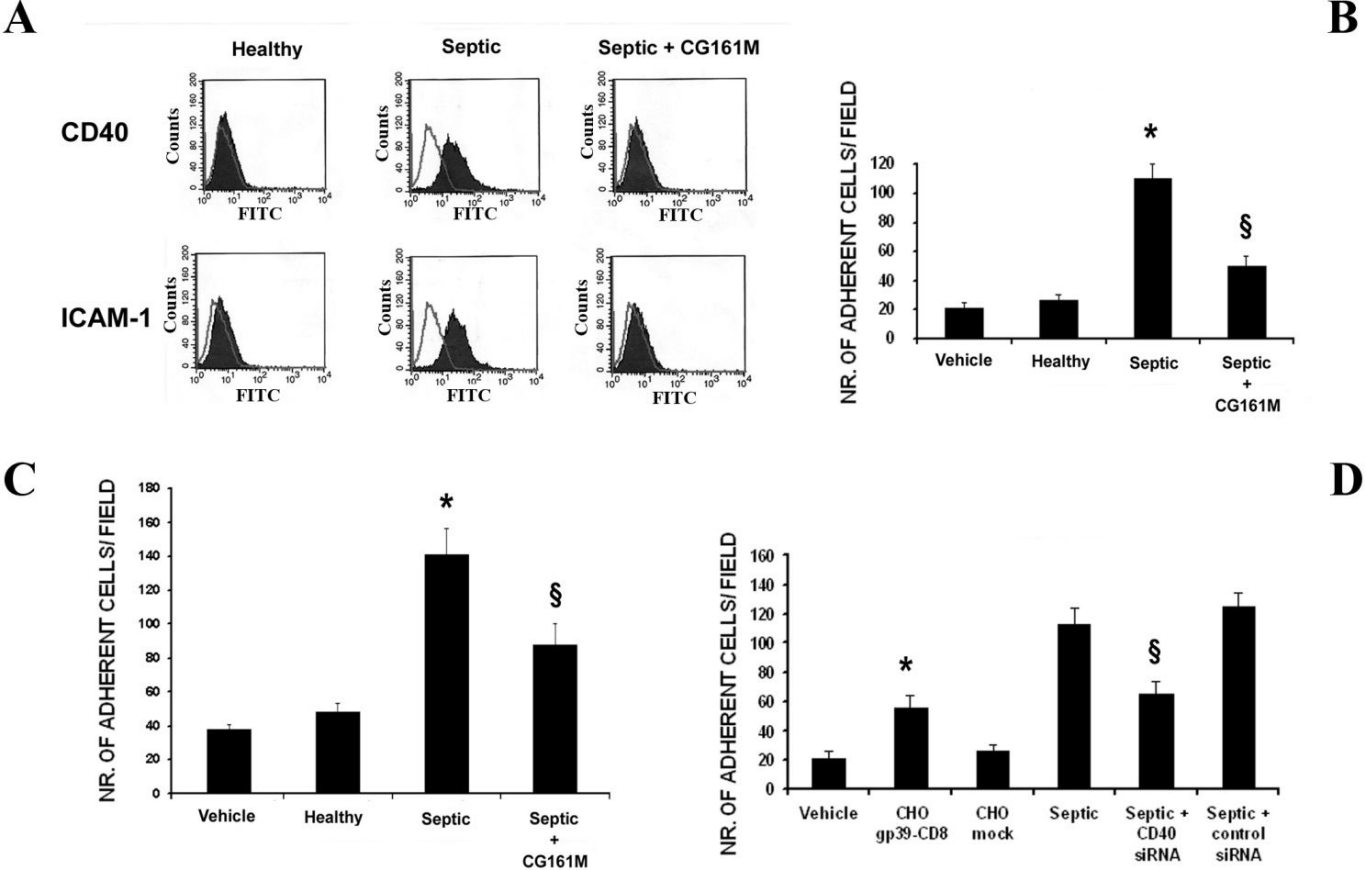
**Evaluation of adhesion molecule expression by TEC and binding of PMN to TEC and HUVEC**. **(a) **FACS analysis of CD40 and inter-cellular adhesion molecule-1 (ICAM-1) expression by tubular epithelial cells (TEC) incubated with healthy plasma, septic plasma or septic plasma after Amberchrom resin adsorption (Septic + CG161 M). Kolomogorov Smirnov statistical analysis was performed. **(b and c) ***In vitro *adhesion assay of freshly purified polymorphonuclear neutrophils (PMN) on **(b) **TEC or **(c) **human umbilical vein endothelial cells (HUVEC) monolayers: Amberchrom resin adsorption significantly reduced septic plasma-induced PMN-TEC and PMN-HUVEC interaction (*P < 0.05 Septic *vs *Healthy; ^§^*P *< 0.05 Septic + CG161 M vs. Septic). **(d) **Effect of the CD40/CD154 pathway on PMN/TEC interaction: PMN adhesion was significantly increased in presence of supernatants collected from chinese hamster ovary (CHO) cells transfected with a cDNA coding for a soluble form of CD154 but not with an empty vector (*P < 0.05 CHO gp39-CD8 vs. Vehicle or CHO mock) and decreased in TEC engineered by siRNA to knock-down CD40 (^§^*P *< 0.05 Septic + CD40 siRNA *vs *Septic or Septic + control siRNA). Data in b and c are expressed as average number ± standard deviation fluorescent cells in 10 different fields (×100 magnification). Analysis of variance with Newmann-Keuls multicomparison test was performed.

Septic plasma induced an increased expression of the costimulatory molecule CD40 and of the adhesion receptor ICAM-1 on TEC surface (Figure 1a), molecules that are both deeply involved in the PMN-TEC interaction [28]. Septic plasma induced a significant increase of PMN adhesion to TEC and to HUVEC in comparison to healthy plasma (Figures 1b and 1c). Plasma adsorption with Amberchrom resin significantly inhibited PMN adhesion on both cell types (Figures 1b and 1c). The decreased expression of ICAM-1 and in particular of CD40 on TEC could account for the reduced PMN adhesion. Indeed, pre-adsorption of septic plasma with Amberchrom resin inhibited the increased TEC expression of CD40 and ICAM-1 (Figure 1a). Moreover, PMN adhesion was increased after incubation of TEC with supernatants of CHO cells transfected with a cDNA coding for a soluble form of CD154 (CD40L), but not with an empty vector (Figure 1d). In addition, a significant decrease of septic plasma-induced PMN adhesion was observed when TEC were transfected by CD40 siRNA but not by control siRNA (Figure 1d).

### Effect of plasma adsorption on TEC apoptosis and cytokine levels

**Figure 2:**
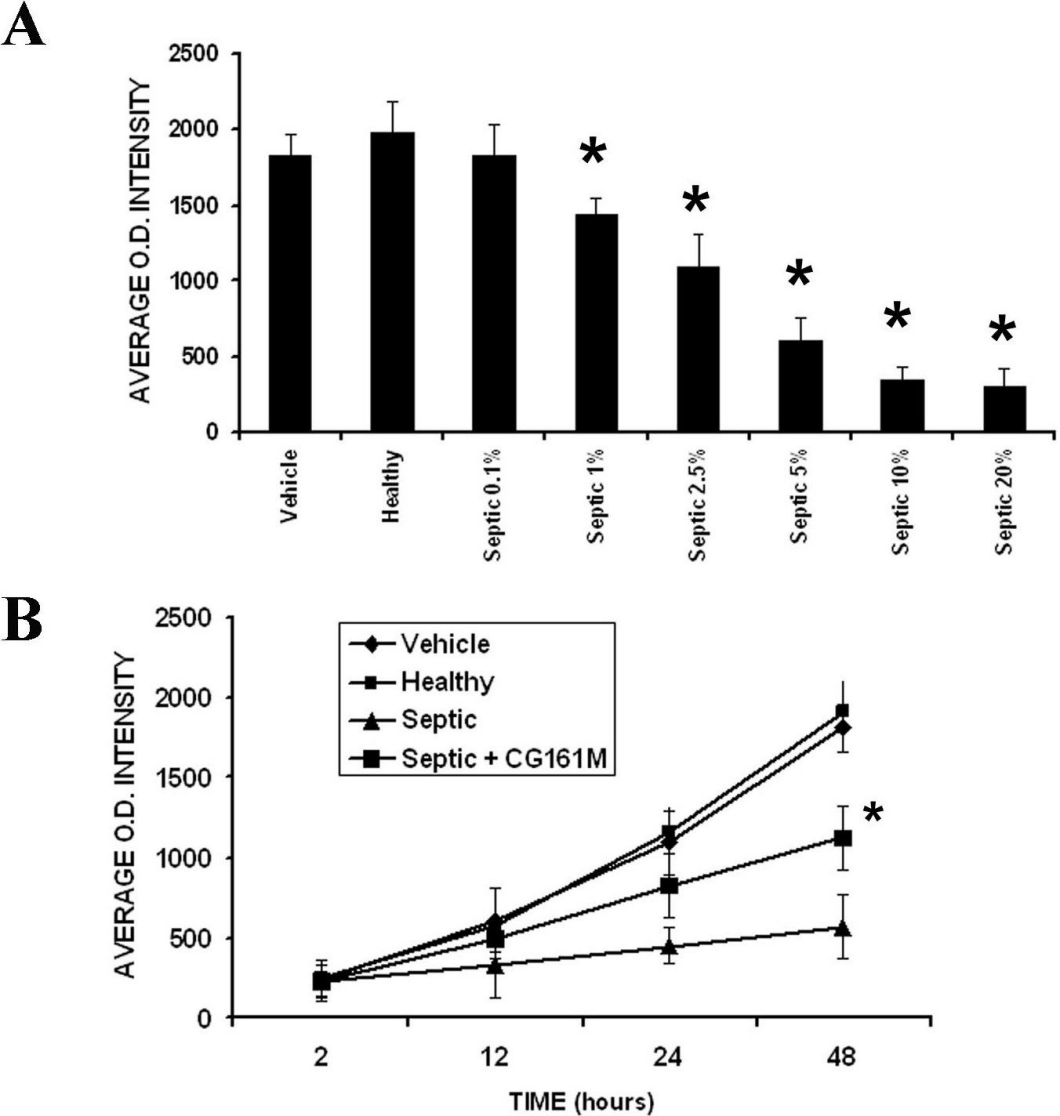
**Protective effect of Amberchrom resin adsorption on septic plasma-induced TEC cytotoxicity**. **(a)** Evaluation of cytotoxicity (XTT-based assay) after incubation of tubular epithelial cells (TEC) for 48 hours with increasing doses of septic plasma diluted in normal culture medium (RPMI 1640). Doses of 1% or more induced a significant decrease of TEC viability (*P < 0.05 Septic 1%, 2.5%, 5%, 10% and 20% vs Healthy plasma). **(b)** Time-course analysis of TEC cytotoxicity (XTT-based assay) induced by 5% septic plasma before and after Amberchrom resin adsorption. TEC treated with pre-adsorbed plasma showed a significant increase of hours at all time points considered (*P < 0.05 Septic + CG161 M vs. Septic at 12, 24 and 48 hours). Data are expressed as average O.D. intensity ± standard deviation. Analysis of variance with Newmann-Keuls multicomparison test was performed.

**Figure 3:**
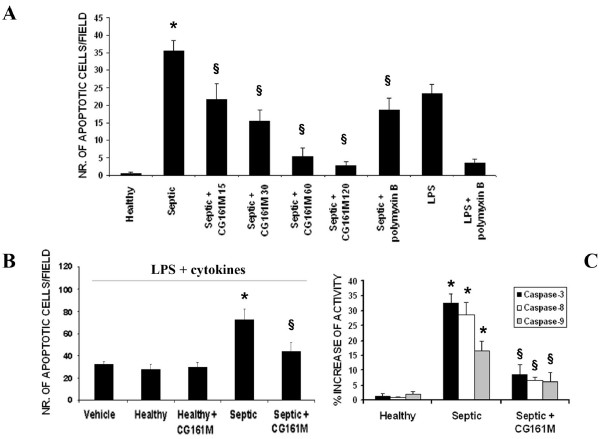
**Significant decrease of septic plasma-induced TEC apoptosis and caspase activation after Amberchrom resin adsorption**. **(a) **Evaluation of tubular epithelial cells (TEC) apoptosis (TUNEL assay) induced by incubation for 48 hours with septic plasma before and after (septic + CG161 M) Amberchrom resin adsorption for 15, 30, 60 or 120 minutes. Septic plasma induced a marked increase of TEC apoptosis (*P < 0.05 Septic *vs *Healthy) that was significantly reduced in presence of plasma subjected to resin adsorption at all times points considered (^§^*P *< 0.05 Septic + CG161 M 15, 30, 60 or 120 minutes vs. Septic). Pre-incubation of septic plasma with 5 μg/ml polymyxin B significantly reduced but not completely abrogated their pro-apoptotic effect on TEC (^§^*P *< 0.05 Septic + polymyxin B *vs *Septic). Lipopolysaccharide (LPS; 20 ng/ml) was used as experimental control. Data are expressed as average number of green fluorescent apoptotic cells ± standard deviation in 10 different fields (×100 magnification). Analysis of variance with Newmann-Keuls multicomparison test was performed. **(b) **Evaluation of TEC apoptosis (TUNEL assay) induced by LPS (30 ng/ml) and inflammatory cytokines (TNF-α 20 ng/ml, IFN-γ 20 ng/ml) after pre-incubation with different plasma. Pre-incubation with septic plasma but not with healthy plasma induced an additive effect on LPS/cytokine-induced TEC apoptosis (*P < 0.05 Septic *vs *Healthy). This effect was significantly decreased after resin adsorption (^§^*P *< 0.05 Septic + CG161M vs. Septic). **(c) **ELISA evaluation of caspase-3, -8 and -9 activities in TEC incubated for 48 hours with control healthy plasma or septic plasma before and after (Septic + CG161M) Amberchrom resin adsorption for 120 minutes. Septic plasma induced a significant increase of all caspase activities (*P < 0.05 caspase-3, -8 and -9 Septic vs. Healthy), whereas Amberchrom resin adsorption significantly reduced plasma-induced caspase activation (^§^*P *< 0.05 caspase-3, -8 and -9 Septic + CG161M *vs *Septic). Results are given as % increase of caspase activities in comparison to unstimulated TEC.

**Figure 4:**
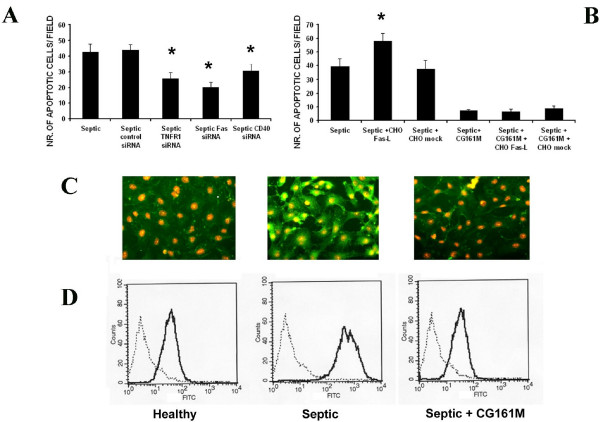
**Protective effect of resin adsorption on septic plasma-induced sensitisation of TEC to death receptor-mediated apoptosis**. **(a) **Evaluation of apoptosis (TUNEL assay) induced by incubation for 48 hours with septic plasma on tubular epithelial cells (TEC) transfected with specific siRNA to knock-down TNFR1, Fas or CD40 expression. The rate of apoptosis was significantly decreased in TEC transfected with all tested siRNA (*P < 0.05 Septic TNFR1 siRNA, Septic Fas siRNA or Septic CD40 siRNA vs. Septic or Septic control siRNA). **(b) **Sensitization of TEC to plasma-induced apoptosis (TUNEL assay) after incubation with supernatants collected from chinese hamster ovary (CHO) cells transfected with a cDNA coding for a soluble form of Fas Ligand (CHO Fas-L) but not with an empty vector (CHO mock) (*P < 0.05 Septic + CHO FasL vs. Septic or Septic + CHO mock). CHO cell supernatants did not influence the apoptotic rate of TEC in presence of plasma pre-adsorbed with the Amberchrom resin. In a and b, data are expressed as average number of green fluorescent apoptotic cells ± standard deviation in 10 different fields (×100 magnification). Analysis of variance with Newmann-Keuls multicomparison test was performed. **(c and d) **Representative immunofluorescence micrographs **(c) **and FACS analysis **(d) **of Fas expression in TEC incubated with healthy plasma or septic plasma before and after (Septic + CG161 M) Amberchrom resin adsorption. In c, nuclei were counterstained by 1 μg/ml propidium iodide (×200 magnification). In d, Kolomogorov Smirnov statistical analysis was performed.

**Figure 5:**
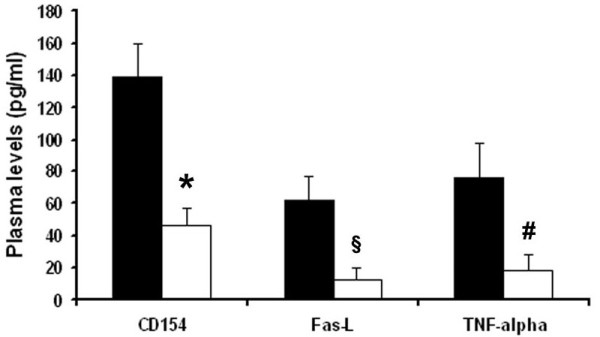
**Significant decrease of cytokine levels in septic plasma after Amberchrom resin adsorption**. ELISA assay of soluble CD154, soluble Fas-L and TNF-alpha levels in plasma collected from septic patients before (dark columns) and after (white columns) Amberchrom resin adsorption for 120 minutes. Resin adsorption induced a significant decrease of all cytokines tested (*P *< 0.05 *CD154, ^§^Fas-L or ^#^TNF-alpha septic + CG161 M vs. septic). Results are expressed as average ± standard deviation. For statistical analysis, t-student test was performed.

Increasing concentrations of plasma derived from patients with sepsis-related AKI induced a significant cytotoxic effect on cultured TEC, as detected by the XTT-based assay after 48 hours incubation (Figure 2a). The cytotoxic effect was absent when TEC were cultured with plasma of healthy volunteers. Septic plasma-induced TEC toxicity was detected after 12 hours incubation and remained significantly higher after 24 and 48 hours with an average 50 to 60% decrease of TEC viability (Figure 2b). In contrast, incubation of TEC with plasma obtained after Amberchrom resin adsorption showed a significant reduction of their cytotoxic activity on TEC at all time points considered (Figure 2b). The cytotoxic effect exerted by septic plasma on TEC was ascribed to the apoptotic cascade pathway. Indeed, as showed by TUNEL assay, exposure of TEC for 48 hours to septic plasma induced a significant increase of apoptosis in respect to healthy plasma (Figure 3a). However, when TEC were cultured for 48 hours in the presence of Amberchrom resin-adsorbed plasma, the apoptotic rate was significantly reduced (Figure 3a). The inhibition of plasma-induced apoptosis was observed after incubation of TEC with samples obtained after 15, 30, 60 and 120 minutes from the beginning of adsorption. The maximal inhibition of plasma-induced apoptosis of TEC was detected with samples obtained after 120 minute adsorption (Figure 3a). LPS (30 ng/ml) was used as a positive control (Figure 3a). Interestingly, the addition in culture of 5 μg/ml polymyxin B significantly reduced but did not completely abolish the pro-apoptotic activity of septic plasma (Figure 3a). These results were confirmed by counting nuclear fragmentation, a typical feature of apoptotic cells, after propidium iodide staining (not shown). Moreover, the pre-incubation with septic plasma induced a significant increase of TEC apoptosis in the presence of LPS and inflammatory cytokines (Figure 3b). This effect was not observed with plasma previously subjected to resin adsorption or with healthy plasma (Figure 3b). In accordance to the TUNEL data, the activities of caspases-3, -8 and -9 were significantly increased in TEC incubated with septic plasma. In contrast, a significant reduction of all caspase activities was observed in TEC cultured in the presence of Amberchrom resin-treated plasma (120 minutes of treatment; Figure 3c). These results suggest that plasma-induced TEC apoptosis was predominantly associated to the activation of the death-receptor pathway induced by soluble mediators. Indeed, the knock-down of TNF-R1, Fas and CD40 in TEC by specific siRNA significantly decreased the pro-apoptotic activity of septic plasma (Figure 4a). We also found that supernatants collected from CHO cells transfected with human Fas-L cDNA induced a significant increase of septic plasma-associated apoptosis (Figure 4b). The apoptotic rate of plasma-treated TEC was not affected by supernatants derived from mock-transfected CHO cells (Figure 4b). These data suggest that septic plasma induced a sensitization of TEC to Fas-mediated apoptosis. Amberchrom resin adsorption abrogated the sensitization of TEC to Fas-mediated apoptosis (Figure 4b). The sensitization of TEC to Fas-mediated apoptosis may be ascribed to the up-regulation of Fas on TEC surface induced by septic plasma that was not observed after Amberchrom resin adsorption (Figures 4c and 4d). In addition, Amberchrom resin adsorption reduced the concentration of pro-apoptotic soluble plasma factors. The high binding capacity of the Amberchrom resin for different inflammatory cytokines was first evaluated in dynamic tests (Table 1). The binding activity of the Amberchrom resin was confirmed by ELISA data on patients' plasma (Figure 5). At study admission, septic patients presented high plasmatic levels of TNF-α, soluble Fas-L and soluble CD40-Ligand (CD154). After 120 minutes absorption by Amberchrom resin, all tested cytokines significantly decreased (Figure 5).

**Table 1:** *In vitro *dynamic test of cytokine adsorption by Amberchrom CG161 M resin

Cytokine	Concentration (pg/ml)	Amount bound (% leakage) (pg/ml adsorbent)	Theoretical binding capacity (pg/ml adsorbent)
TNF-α	675 ± 72	4999 (5%) ± 1703	200,980 ± 53,607
IL-1-β	331 ± 1	61,439 (5%) ± 10,459	116,842 ± 24,707
IL-6	334 ± 59	35,729 (5%) ± 8149	912,776 ± 200,096
IL-8	340 ± 186	322 (12%) ± 161	48,940 ± 26,089
IL-10	464 ± 143	69,187 (5%) ± 31,112	972,880 ± 61,006

The conditions used for the dynamic studies on Amberchrom CG161 M resin are extensively described in the Materials and Methods. Briefly, the resin columns were perfused at a low linear velocity (0.30 ml/min). The theoretical binding capacity of the resins for individual cytokines which was calculated by extrapolation of the increase in cytokine concentration at the column outlet over time only gives a rough estimation of the actual maximal binding capacity.

### Effect of resin adsorption on functional TEC alterations

**Figure 6:**
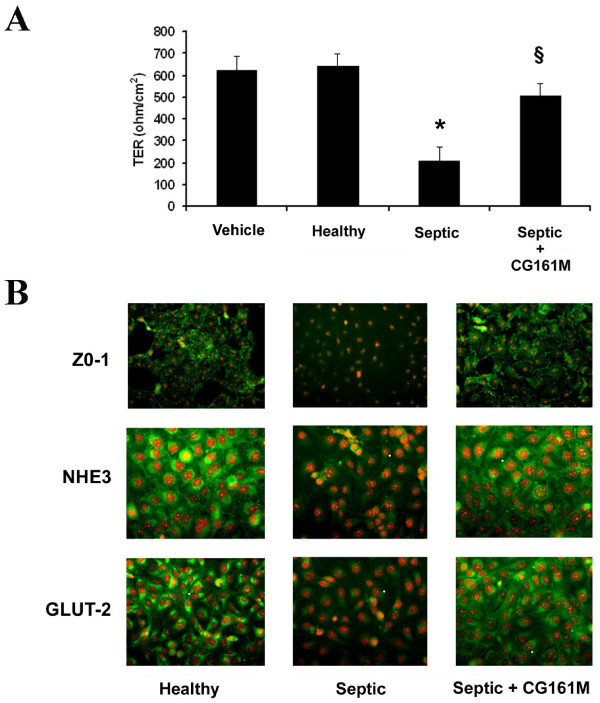
**Effect of resin adsorption on septic plasma-induced alteration of polarity and expression of TEC transporters**. **(a) **Evaluation of tubular epithelial cells (TEC) polarity expressed as trans-epithelial electrical resistance (TER). Septic plasma induced a significant decrease of TER (*P < 0.05 Septic vs. Healthy or Vehicle) that was inhibited by Amberchrom resin adsorption (^§^*P *< 0.05 Septic + CG161 M *vs *Septic). Data are expressed as average TER values (ohm/cm^2^) ± standard deviation. Results were normalized for the membrane area of transwell used in the test. Analysis of variance with Newmann-Keuls multicomparison test was performed. **(b) **Representative immunofluorescence micrographs of the expression of the tight junction protein zonula occludens-1 (ZO-1), the sodium channel NHE3 and the glucose transporter GLUT-2 in TEC cultured with control healthy plasma or septic plasma before and after (Septic + CG161 M) Amberchrom resin adsorption. Nuclei were counterstained by 1 μg/ml propidium iodide (magnification ×200).

**Figure 7:**
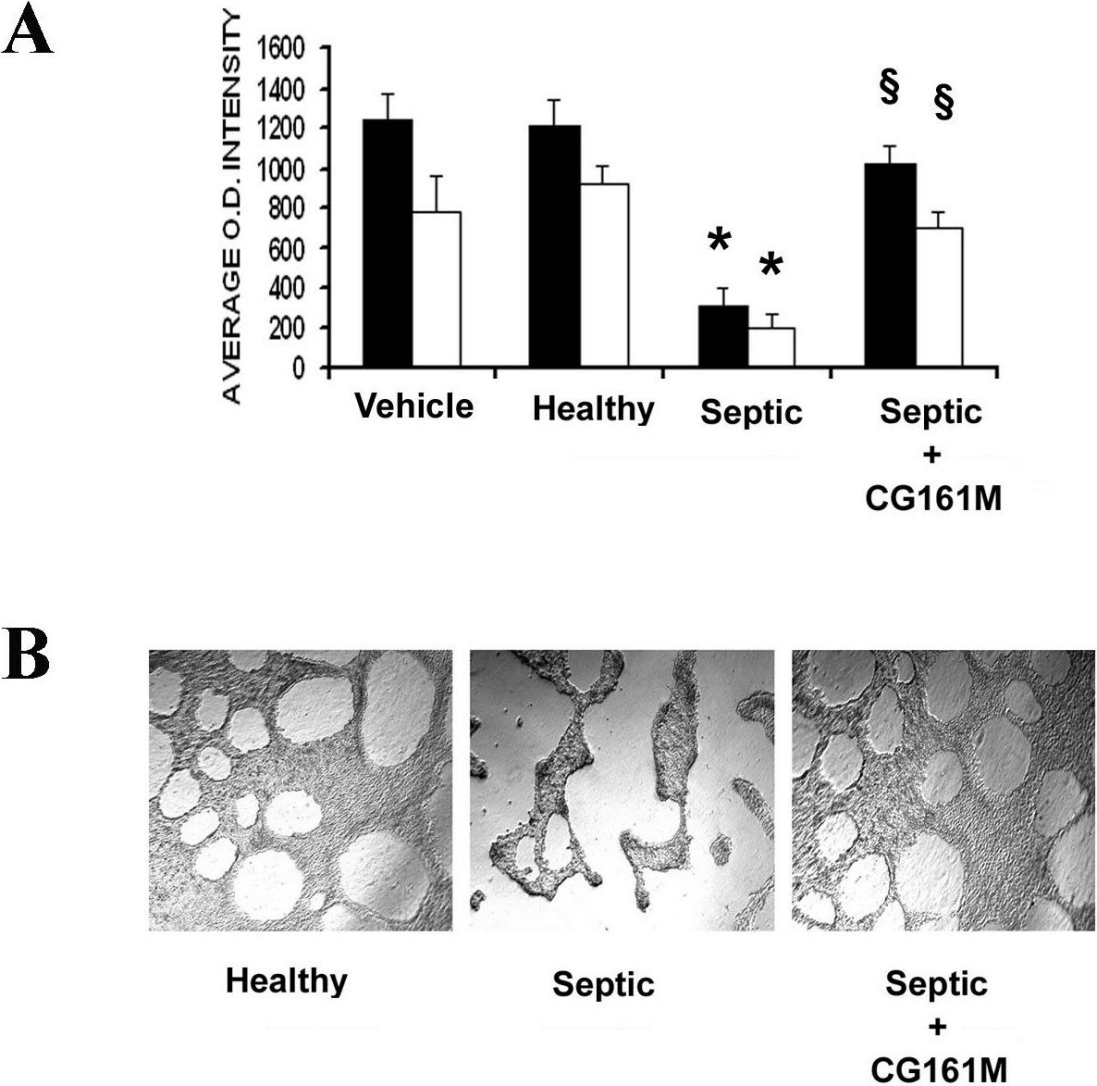
**Effect of resin adsorption on septic plasma-induced alteration of adhesion to matrixes and TEC morphogenesis**. **(a) **In vitro adhesion assay of tubular epithelial cells (TEC) to extracellular matrixes. Septic plasma induced a significant decrease of adhesion of TEC to Type IV collagen/fibronectin (dark columns) or Matrigel (white columns) (*P < 0.05 Septic vs. Healthy or Vehicle). In contrast, Amberchrom resin adsorption significantly decreased the inhibitory effect of septic plasma on TEC adhesion to all matrixes tested (^§^P < 0.05 Septic + CG161 M vs. Septic). Data are expressed as average O.D. intensity ± standard deviation. Analysis of variance with Newmann-Keuls multicomparison test was performed. **(b)** Representative micrographs of TEC morphogenesis after 48 hours culture on Matrigel-coated plates in presence of control healthy plasma or septic plasma before and after (septic + CG161 M) Amberchrom resin adsorption.

**Figure 8:**
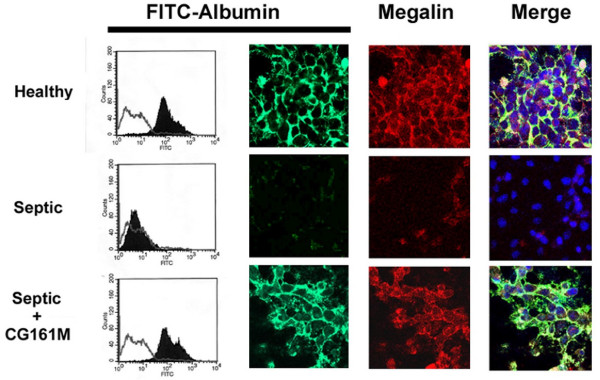
**Effect of resin adsorption on albumin internalization and expression of megalin by TEC**. Representative FACS and confocal microscopy analysis of FITC-albumin uptake (green fluorescence) and megalin expression (red fluorescence) in tubular epithelial cells (TEC) incubated with control healthy plasma or septic plasma before and after (Septic + CG161 M) Amberchrom resin adsorption. For FACS analysis, Kolomogorov Smirnov statistical analysis was performed. In merge images, nuclei were counterstained by 0.5 μg/ml Hoechst.

Septic plasma significantly reduced TER, an indicator of TEC polarity. This effect was abrogated in the presence of Amberchrom resin-treated plasma (Figure 6a). Further evidence for the maintenance of TEC polarity and function came from the observation that Amberchrom resin abrogated the down-regulation of the tight junction protein ZO-1, proximal tubular cell sodium transporter NHE3 and glucose transporter GLUT-2, which were all induced by septic plasma (Figure 6b). In addition, the reduced adhesion of TEC to the extracellular matrixes fibronectin/type IV collagen and Matrigel observed in the presence of septic plasma was significantly inhibited after Amberchrom resin adsorption (Figure 7a). TEC cultured on Matrigel-coated plates showed a typical morphology characterized by early scattering and branching morphogenesis that was reduced after incubation with septic plasma (Figure 7b). In contrast, TEC morphogenesis was not affected by incubation with Amberchrom-adsorbed plasma (Figure 7b). Moreover, we found that septic plasma induced the down-regulation of the endocytic receptor megalin, a molecule involved in tubular re-adsorption of filtered proteins (Figure 8). The decreased expression of megalin was not observed in the presence of Amberchrom resin-treated plasma (Figure 8). This phenomenon was probably responsible for the preserved ability of TEC to internalize FITC-labeled albumin (Figure 8).

## Discussion

The results of the present study showed that septic plasma induced TEC injury, which was abrogated by non-selective removal from the plasma of factors responsible for PMN-TEC adhesion and for apoptosis and altered polarity of TEC.

The mechanisms responsible for AKI in the course of sepsis are not fully elucidated. It has been hypothesized that inflammatory factors present in the circulation or locally produced by resident kidney cells may have an active role in the pathogenesis of tissue damage [8,10,29]. Indeed, patients with AKI have elevated plasmatic levels of inflammatory cytokines and high levels of IL-6 and IL-8 are associated with an increased risk of mortality [30,31].

In the present study, septic plasma was adsorbed with Amberchrom CG161 M, a rigid, hydrophobic, highly cross-linked microreticular adsorbent polymer. Its high binding capacity depends on the relatively small pore structure (median pore size 150 to 200 Å, exclusion limit 70 kDa) and high internal surface area (900 m^2^/g). The mean particle size of this polymer is approximately 75 μm, which is convenient for achieving a balance of diffusional access and flow [20]. We performed dynamic tests as confirmation of the high binding capacity of the Amberchrom resin for different inflammatory cytokines present simultaneously at very high concentrations [20]. Although in this study we focused on the cytokine adsorption, one may expect that other proteins can bind to the resin as the hydrophobic polymer exhibits a non-selective affinity with respect to proteins depending on the exposure of interacting domains. However, experimental and clinical evidence has suggested that non-selective removal of molecules in severe sepsis is beneficial [32,33].

In the very early events of sepsis-induced AKI, the adhesion of PMN to TEC may contribute to the pathogenesis of tissue injury [34]. This process is mediated by adhesion molecules such as CD40 and ICAM-1 that are up-regulated by inflammatory cytokines [35,36]. Indeed, ICAM-1-deficient mice are protected from experimental sepsis-induced AKI [37]. CD40 also plays a crucial role in the innate response and its inhibition is related to a decrease of mortality in experimental septic models [38]. Moreover, the activation of the CD40/CD154 pathway in TEC induces a pro-fibrotic and pro-inflammatory state [39,40]. We found that after Amberchrom resin adsorption, septic plasma lost the capacity to up-regulate ICAM-1 and to activate the CD40/CD154 pathway on cultured human TEC. This effect may be ascribed to the removal of soluble CD154 from septic plasma. In the course of sepsis, activated platelets and leukocytes may release high amounts of soluble CD154 from their surface that interacts with the CD40 expressed by TEC and other target cells [41,42]. In this setting, the removal of soluble CD154 as well as other inflammatory mediators by resin adsorption may lead to the inhibition of PMN adhesion to TEC.

It has been suggested that apoptosis plays a key role in the pathogenetic mechanisms of sepsis-related tissue injury including AKI [5,7,43-45]. However, a systematic review of the histopathological findings in septic AKI reported a normal histology in the majority of cases [46]. As apoptosis is a reversible process and apoptotic cells are rapidly removed by phagocytic cells it may be difficult to detect tubular apoptotic cells in the histological specimens.

Tubular injury is not the mere result of renal hypoperfusion or cortico-medullary redistribution of blood flow [5,47]. A direct toxic effect of circulating or locally-produced inflammatory cytokines and LPS has been postulated as the main cause of sepsis-related AKI [7-10]. We found that TEC apoptosis was induced by incubation with septic plasma through the activation of Fas and caspases. These events were all abrogated following Amberchrom resin adsorption of septic plasma. Indeed, we observed a significant reduction of TNF-α, soluble Fas-L and soluble CD154 in septic plasma after incubation with the Amberchrom resin. The relevance of TNF-α, soluble Fas-L and soluble CD154 in inducing TEC injury was confirmed by the observed decrease of apoptosis in TEC engineered to knock-down Fas, CD40 or TNF-R1 by specific siRNA. These results suggest that the unselective removal of circulating inflammatory substances by Amberchrom resin may be responsible for the inhibited activation of the death receptor-mediated apoptotic pathway in TEC.

With the purpose of evaluating the protective effect of resin adsorption on the extension of sepsis-associated AKI after the initial insult, we performed a set of experiments aimed at evaluating the role of a 'priming activity' of septic plasma on TEC apoptosis. We found that the pre-incubation of tubular cells with septic plasma provoked an additional effect on apoptosis induced by LPS and inflammatory cytokines. This effect was decreased after adsorption of septic plasma by CG161 M resin. These results suggest that plasma adsorption may limit the continued renal injury sustained by these mediators.

Despite their pro-apoptotic effect, inflammatory cytokines are also known to alter actin cytoskeleton distribution and integrin cell-matrix interaction *via *a nitric oxide-dependent mechanism [48,49]. These events lead to the shedding of viable or dead cells into the tubular lumen, causing a possible obstruction to urine flow and back-leakage of fluid in the interstitial spaces [50] associated with alteration of cell polarity, a biological function of epithelial cells essential to maintain a correct electrolyte distribution in distinct fluid-filled compartments [51]. In experimental models of sepsis, a dysfunction at tight junction level in different organs has been observed [52,53]. Indeed, inflammatory cytokines induce tight junction dysfunction in intestinal, pulmonary and hepatic epithelial cells, an event probably ascribed to an increased inducible nitric oxide synthase activity [54-56]. In addition, it has been shown that during severe inflammation renal sodium, chloride, urea and glucose transporters are significantly down-regulated via a cytokine-dependent mechanism [11-13]. Here we show that Amberchrom resin adsorption inhibited the septic plasma-induced decrease of TER and the down-regulation of the tight junction protein ZO-1, sodium channel NHE3 and glucose transporter GLUT-2. Furthermore, the loss of TEC polarity was associated to a redistribution of molecules typically expressed on the apical or basolateral surface [51]. This effect may be responsible for the decreased adhesion of TEC to extracellular matrixes as well as for the altered morphogenesis. All these biological effects were inhibited by treatment of plasma with the Amberchrom resin, suggesting a protective effect related not only to the inhibition of TEC apoptosis, but also to the preservation of cell polarity and function.

Microalbuminuria is a typical finding in septic patients [57]. Urinary loss of proteins may be related to an increased permeability of the glomerular filtration barrier [58]. However, injured TEC may contribute to proteinuria through the impairment of reabsorption. In this setting, megalin is an endocytic receptor involved in the reabsorption of proteins with different molecular weight, including albumin [59]. Megalin-deficient mice are characterized by the development of low molecular weight proteinuria [60]. We found that plasma from septic patients induced the loss of megalin from TEC and inhibited FITC-labelled albumin reabsorption. Amberchrom resin adsorption prevented the loss of megalin expression and of albumin uptake by TEC induced by septic plasma. These results also indicate a possible protective effect of resin adsorption on the maintenance of protein uptake by injured TEC.

The occurrence and relevance of apoptosis and of increased tubular permeability in human sepsis-associated AKI needs to be further critically evaluated. However, we may hypothesize that plasma circulating in the peritubular capillaries may enhance PMN adhesiveness to endothelial, promote vascular permeability and later induce a direct injury of tubular cells leading to cell apoptosis and altered cell polarity and function. It is reasonable to think that these changes occur at a very early stage after the primary insult.

## Conclusions

The results of this study showed that septic plasma induced different injurious effects on cultured TEC by favouring PMN adhesion, inducing cell apoptosis and altering cell polarity and function. All these biological effects are related to the presence of circulating inflammatory mediators that can be efficiently removed by unselective resin adsorption with a consequent limitation of tubular cell injury. Whether non-selective removal of cytokines may have a protective or therapeutic role in human sepsis-associated AKI is an attractive possibility which needs further investigation.

## Key messages

• Circulating factors present in plasma of septic patients induce granulocyte adhesion, apoptosis and alter polarity in tubular cells, biological events involved in the pathogenesis of sepsis-associated AKI.

• Resin adsorption inhibits septic plasma-induced granulocyte adhesion to endothelial and tubular cells mediated by the adhesion receptor ICAM-1 and by the costimulatory molecule CD40.

• Resin adsorption inhibits the apoptosis of tubular cells induced by septic plasma which is dependent on the activation of caspase-3, -8, -9 and of the Fas/death receptor-mediated signalling pathways.

• Resin adsorption inhibits the alteration of tubular cell polarity, morphogenesis, protein uptake and the down-regulation of the tight junction molecule ZO-1, of the sodium transporter NHE3, of the glucose transporter GLUT-2 and of the endocytic receptor megalin all induced by septic plasma.

• The inhibition of septic plasma induced-tubular cell injury after resin adsorption is associated with the decrease of soluble mediators involved in inflammation and apoptosis such as TNF-α, Fas-L and CD40-L.

## Abbreviations

AKI: acute kidney injury; BSA: bovine serum albumin; CD40-L: CD40-Ligand; CHO: chinese hamster ovary; ELISA: enzyme-linked immunosorbent assay; Fas-L: Fas-Ligand; FCS: fetal calf serum; GLUT-2: glucose transporter-2; HUVEC: human umbilical vein endothelial cells; ICAM-1: inter-cellular adhesion molecule-1; ICU: intensive care unit; IFN-γ: interferon-γ; IL: interleukin; LPS: lipopolysaccharide; NHE3: sodium-hydrogen exchanger-3; PBS: phosphate-buffered saline; PMN: polymorphonuclear neutrophils; pNA: p-nitroanilide; RRT: renal replacement therapy; RT-PCR: reverse-transcription polymerase chain reaction; SD: standard deviation; SOFA: Sequential Organ Failure Assessment; TEC: tubular epithelial cells; TER: trans-epithelial electrical resistance; TNF-α: tumor necrosis factor-α; TNF-R1: tumor necrosis factor receptor 1; ZO-1: zonula occludens-1.

## Competing interests

CT is a full-time employee of Fresenius Medical Care Deutschland GmbH. The Company will neither gain nor lose from the publication of this manuscript at present or in the future.

Until November 2002, CT was a full-time employee of Bellco S.p.A that owns the herein cited patent of which Ciro Tetta, Mary Lou Wratten and Luisa Sereni are the inventors "wo99109637 1-2305: Method for extracorporeal removal of toxins in particular cytokines, particularly for treating patients affected with acute organ failure". Bellco S.p.A. commercialyzes an extracorporeal device based on this patent, named as coupled plasma filtration adsorption (CPFA). Bellco S.p.A. could neither lose or gain if this manuscript is published because the adsorption material is different from the commercialized one.

The other authors declare that they have no competing interests.

## Authors' contributions

VC and CT conceived the study, analyzed and interpreted the data and elaborated the manuscript. MDC, DC and CR performed the enrollment of patients in the study and carried out the analysis of clinical and biochemical parameters. VW and CT established the *in vitro *model of resin adsorption and performed the dynamic tests on cytokine removal. VC, CL, FF and SB established the *in vitro *model of tubular cell injury and performed the *in vitro *assays. LB developed the plasmid vectors used in the study. LB, CR and GPS participated in the design of the study and in the interpretation of the results. GC conceived and supervised the study, analyzed and interpreted the data and corrected the final version of the manuscript. All authors read and approved the final version of the manuscript.
